# Telephone Consultation for Improving Health of People Living with or at Risk of HIV: A Systematic Review

**DOI:** 10.1371/journal.pone.0036105

**Published:** 2012-05-17

**Authors:** Michelle H. M. M. T. van Velthoven, Lorainne Tudor Car, Josip Car, Rifat Atun

**Affiliations:** 1 Global eHealth Unit, Department of Primary Care and Public Health, Imperial College London, London, United Kingdom; 2 Imperial College Business School, Imperial College London, London, United Kingdom; McGill University Health Centre, - McGill University, Canada

## Abstract

**Background:**

Low cost, effective interventions are needed to deal with the major global burden of HIV/AIDS. Telephone consultation offers the potential to improve health of people living with HIV/AIDS cost-effectively and to reduce the burden on affected people and health systems. The aim of this systematic review was to assess the effectiveness of telephone consultation for HIV/AIDS care.

**Methods:**

We undertook a comprehensive search of peer-reviewed and grey literature. Two authors independently screened citations, extracted data and assessed the quality of randomized controlled trials which compared telephone interventions with control groups for HIV/AIDS care. Telephone interventions were voice calls with landlines or mobile phones. We present a narrative overview of the results as the obtained trials were highly heterogeneous in design and therefore the data could not be pooled for statistical analysis.

**Results:**

The search yielded 3321 citations. Of these, nine studies involving 1162 participants met the inclusion criteria. The telephone was used for giving HIV test results (one trial) and for delivering behavioural interventions aimed at improving mental health (four trials), reducing sexual transmission risk (one trial), improving medication adherence (two trials) and smoking cessation (one trial). Limited effectiveness of the intervention was found in the trial giving HIV test results, in one trial supporting medication adherence and in one trial for smoking cessation by telephone.

**Conclusions:**

We found some evidence of the benefits of interventions delivered by telephone for the health of people living with HIV or at risk of HIV. However, only limited conclusions can be drawn as we only found nine studies for five different interventions and they mainly took place in the United States. Nevertheless, given the high penetration of low-cost mobile phones in countries with high HIV endemicity, more evidence is needed on how telephone consultation can aid in the delivery of HIV prevention, treatment and care.

## Introduction

HIV/AIDS contributes significantly to the global burden of disease. In 2010, 34 million people were living with HIV (PLHIV), including an estimated 2.7 million people who had become newly infected with HIV that year. The vast majority of these infections occur in low and middle-income African countries with weak health systems [1]. The AIDS epidemic is projected to continue over the next few decades and the number of PLHIV will increase [2].

In 2003 only 400,000 PLHIV in low and middle-income countries were receiving antiretroviral treatment (ART) [3]. By 2011 this number had increased to 7.4 million, although approximately a further seven million people who needed treatment did not have access to ART [4]. The rapid growth in the number of people receiving ART means health systems must continue to provide acute life-saving care for those with advanced AIDS, while also providing chronic services to expanding cohorts of patients who are doing well on ART and preventive interventions for the populations at risk [5]. Low cost, effective interventions are needed if prevention and treatment programmes are to be sustained [6].

Globally, most people have access to a phone, either a telephone or mobile phone [7]. While landlines are mainly used in the developed world, mobile phones are widespread around the world. In 2011 there were almost six billion mobile subscriptions worldwide, with an estimated 3.8 billion subscriptions in low and middle-income countries [7]. There is huge potential for phone interventions to improve access to healthcare and to strengthen communication between healthcare workers and patients [8]. Organisations such as the World Health Organization have included scaling up telecommunications technologies for healthcare delivery in resource-limited healthcare settings as one of their priorities [9].

Mobile phone messaging is very popular and shows promise as a simple and cost-effective means of communication in healthcare. However, its use is limited by the short space available in a message and illiteracy of people. Voice calls can overcome these barriers and are used for a broad spectrum of healthcare interventions at low cost, including substitution of traditional face-to-face contacts with healthcare professionals [10]. Telephone consultation is used to manage patients by giving advice or by referral to other services [10], [11]. Telephones can also be used for peer support [12] or for delivering complex interventions such as behavioural or mental health interventions [13], [14]. Compared to face-to-face care, telephone care can reduce costs, save travel and waiting time and improve access to care, as well as enable healthcare workers to consult with more patients within a given time [11], [15]. Structured telephone support has shown to be effective in reducing the risk of mortality and hospitalisations in patients with chronic heart failure [16]. However, healthcare workers need to be appropriately trained to deliver high quality care by telephone [17].

AIDS is now a long-term condition requiring treatment adherence and regular medical care, but one which remains stigmatized. In a context of rising numbers of people requiring ART, concerns about resistance and the high cost of second line regimens have highlighted the importance of adherence [18]. Using telephones in the management of AIDS can help address issues of compliance and confidentiality, for example in vulnerable populations such as sex workers or those who are substance dependent [13]. Helplines have been used both in developed [19] and in developing countries [20] in the management of AIDS. Telephone consultation has been successfully used to provide HIV test results [21], psychotherapy [13], disease management [22], HIV prevention [23], [24], and to improve ART adherence [25].

Telephones offer the potential to improve the health of PLHIV cost-effectively and to reduce the burden on affected people and health systems. However, evidence about the feasibility and effectiveness of using telephones in HIV/AIDS care is inconclusive. This review synthesizes evidence from randomised controlled trials (RCTs) investigating the effectiveness of telephone consultation. It includes trials of consultations using the voice function of the telephone or mobile phone in testing and treatment of PLHIV as well as people at risk of HIV infection and in HIV prevention.

## Methods

We followed the standard methods developed by The Cochrane Collaboration as described in The Cochrane Handbook for Systematic Reviews of Interventions [26]. Briefly summarized this methodology includes the following: defining the review question and developing criteria for inclusion and exclusion of studies; a high sensitivity search for studies with the Cochrane filter for RCTs; search, selection and data extraction (using standardised forms) by two review authors independently; assessing risk of bias using the Cochrane tool and considering meta-analysis when appropriate or a narrative assessment.

### Criteria for Considering Studies for this Review

We included RCTs of interventions with telephones or mobile phones for PLHIV and people at risk of HIV infection. For the control group we considered face-to-face consultations, usual care and groups who only received the outcome assessments. We included studies using landlines or mobile phones for delivering voice calls. Studies using mobile phone messaging (SMS, MMS) were excluded, as this would make the review too diverse (we report on this in an upcoming review).

### Outcome Measures

To assess the effectiveness of interventions we included studies with a range of primary outcomes: clinical outcomes (e.g. immunologic and virologic outcomes, depressive and psychiatric symptoms), health behaviour outcomes (e.g. adherence to treatment, smoking, risky behaviour, disease coping) and outcomes related to healthcare delivery (e.g. proportion of people receiving test results, number of missed appointments). We considered the following secondary outcomes: AIDS-related mortality, social outcomes (e.g. support from family and friends, loneliness), adverse outcomes (e.g. increased out-of -hour’s contact), cost outcomes (e.g. costs, cost-effectiveness) and quality of life. Outcomes of participants receiving telephone interventions were compared with outcomes of participants assigned to the control group.

### Search Methods for Identification of Studies

The search strategy can be found in Table 1. It has three parts: (1) search terms specific to telephone interventions, (2) search terms related to HIV and AIDS and (3) the RCT search filter developed by The Cochrane Collaboration, detailed in The Cochrane Handbook [26]. We consulted a librarian for developing the MEDLINE search strategy and we translated this search for the other databases. We did not limit our search by language or publication year.

**Table 1:** Search strategy MEDLINE.

Number	Search terms
*#*1	phone*
*#*2	telephon*
*#*3	telefon*
*#*4	telephone intervention
*#*5	call
*#*6	calls
*#*7	calling
*#*8	telemedicine
*#*9	teleconsult*
*#*10	telecounsel*
*#*11	Telephone[MeSH]
*#*12	Hotlines[MeSH]
*#*13	Cellular phone[MeSH]
*#*14	#1 OR #2 OR #3 OR #4 OR #5 OR #6 OR #7 OR #8 OR #9 OR #10 OR #11 OR #12 OR #13
*#*15	HIV Infections[MeSH]
*#*16	HIV[MeSH]
*#*17	hiv[tw]
*#*18	hiv-1*[tw]
*#*19	hiv-2*[tw]
*#*20	hiv1[tw]
*#*21	hiv2[tw]
*#*22	hiv infect*[tw]
*#*23	human immunodeficiency virus[tw]
*#*24	human immunedeficiency virus[tw]
*#*25	human immune-deficiency virus[tw]
*#*26	((human immun*) AND(deficiency virus[tw]))
*#*27	“Acquired Immunodeficiency Syndrome”[MeSH]
*#*28	acquired immunodeficiency syndrome[tw]
*#*29	acquired immunedeficiency syndrome[tw]
*#*30	acquired immuno-deficiency syndrome[tw]
*#*31	acquired immune-deficiency syndrome[tw]
*#*32	((acquired immun*) AND (deficiency syndrome[tw]))
*#*33	#15 OR #16 OR #17 OR #18 OR #19 OR #20 OR #21 OR #22 OR #23 OR #24 OR #25 OR #26 OR #27 OR #28 OR #29 OR #30 OR #31 OR #32
*#*34	randomized controlled trial [pt]
*#*35	controlled clinical trial [pt]
*#*36	randomized [tiab]
*#*37	placebo [tiab]
*#*38	drug therapy [sh]
*#*39	randomly [tiab]
*#*40	trial [tiab]
*#*41	groups [tiab]
*#*42	#34 OR #35 OR #36 OR #37 OR #38 OR #39 OR #40 OR #41
*#*43	animals [mh] NOT humans [mh]
*#*44	#42 NOT #43
*#*45	*#* 14 AND *#* 33 AND *#* 44

In July 2010 we searched the following electronic databases: MEDLINE, EMBASE, The Cochrane Central Register of Controlled Trials (CENTRAL, *The Cochrane Library*), PsycINFO, ISI Web of Science, Cumulative Index to Nursing & Allied Health, Dissertation Abstracts International, The World Health Organization’s Global Health Library and CABI (CAB abstracts). We searched online trial registers for ongoing and recently completed studies (Current Controlled Trials: www.controlled-trials.com). We searched the following grey literature databases: OpenSIGLE (System for Information on Grey Literature) and The Healthcare Management Information Consortium (HMIC) database as well as two conference databases: Conference on Retroviruses and Opportunistic Infections (CROI) and International AIDS Society (IAS). A Google Scholar search resulted in a large number of hits (142,000), therefore we screened the first 500 results.

### Data Collection and Analysis

We merged the search results across databases and removed duplicate records with Endnote X4 [27]. Two authors (MHMMTvV and LTC) independently examined titles and abstracts for inclusion criteria and discussed eligibility. We retrieved full texts for studies which seemed relevant and studies for which eligibility remained unclear. The authors discussed which studies met the inclusion criteria and therefore had to be included. We independently extracted data for study methods, participants, interventions, outcomes, results and miscellaneous information using standardized sheets. We assessed bias in included studies using the Cochrane domains [26] as a template for the following domains: sequence generation, allocation sequence concealment, blinding, incomplete outcome data, whether the study addressed attrition, reasons for attrition and how this was handled during analysis, selective outcome reporting, and other potential biases. These other potential biases relevant for this review were: comparability of the intervention and control group characteristics at baseline, imbalance of outcome measures at baseline and protection against contamination.

Statistical pooling of results was not possible because of the extensive heterogeneity of the trials. Therefore, we present a narrative overview of studies in which we systematically describe each included study according to design, setting, participants, control, outcomes and results and group the studies according to intervention type.

## Results

### Search Results

**Figure 1:**
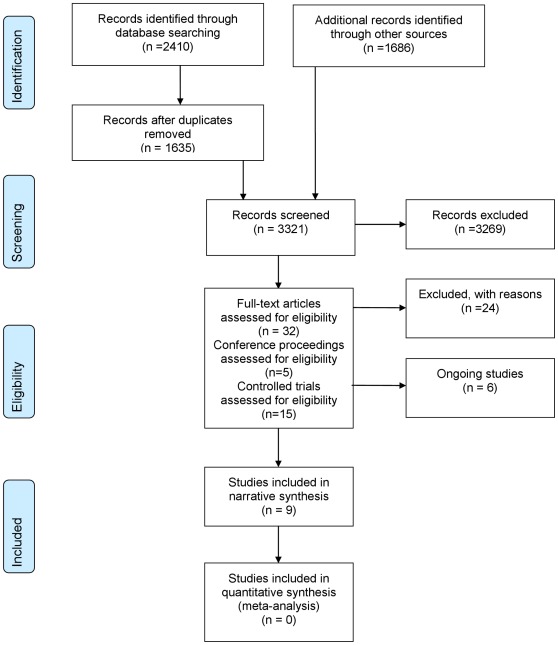
PRISMA (Preferred Reporting Items for Systematic Reviews and Meta-Analyses) flow diagram.

The literature searches yielded 3321 citations. Screening titles and abstracts resulted in 32 records. We included 13 articles representing nine RCTs for which detailed information can be found in the Tables 2, 3 and 4 [28], [29], [30], [31], [32], [33], [34], [35], [36]. We found two reports of the same study for Eaton 1995 [30], [37], Ransom 2008 [33], [38], Stein 2007 [32], [39] and Vidrine 2006 [36], [40]. In addition, we identified six ongoing trials. We excluded 19 studies after full text review [41], [42], [43], [44], [45], [46], [47], [48], [49], [50], [51], [52], [53], [54], [55], [56], [57], [58], [59] and five abstracts [60], [61], [62], [63], [64]. Reasons for exclusion were: not an RCT (n = 5); telephone was only part of the intervention and not evaluated separately (n = 4); missing data (n = 4); participants neither had HIV/AIDS nor were tested for HIV (n = 3); no intervention using telephones was being studied (n = 3); descriptive study (n = 2); no control arm (n = 2). An adapted PRISMA (Preferred Reporting Items for Systematic Reviews and Meta-Analyses) flow-diagram of study selection can be found in Figure 1 [65].

**Table 2:** Overview of HIV testing and HIV prevention studies.

Study	Setting	Participants	Intervention (I)	Control (C)	Outcomes (O)	Results
*HIV testing*						
Tsu 2002 [28]	Portland, Oregon, US.	Homeless and high-risk youth requesting HIV testing.	*Content:* Giving the option of face-to-face or telephone notification of HIV test results. *Delivered by:* ‘trained counsellors’; medical and public health students and clinical staff. *N:* 184.	Usual care group, required to receive test results face-to-face at the clinic. *N:* 168.	O.1) Proportion of participants receiving HIV test results.	O.1) I: 57.6%, C: 37.1%. Significant difference between groups in favour of I (odds ratio = 2.264, 95% confidence interval [1.445–3.547] P<0.001).
*HIV prevention*						
Rotheram-Borus 2004 [29]	AIDS clinics, community-based sites and solicitation in newspapers, conferences, etc. in Los Angeles, San Francisco, and New York, US.	Substance using HIV-positive youth.	*Content:* Telephone calls aiming to reduce HIV transmission. *Duration:* 18 sessions (three modules of six sessions). *Delivered by:* licensed therapists or clinical social workers (mainly women). *N:* 59.	C.1.In person intervention group *N:* 61. C.2.Delayed intervention group (only receiving the outcome assessments) *N:* 55.	O.1) Sexual risk behaviour. O.2) Substance use. O.3) Adherence to antiretroviral treatment regimen. O.4) Number of missed appointments. O.5) Brief Symptom Inventory score (emotional distress). *Time points:* three months, six months, nine months and 15 months.	O.1) Proportion of protected acts with HIV-negative partners. I. Decrease from 75% at baseline to 65% at 15 months. C1. Increase from 53% at baseline to 73% at 15 months. Significant difference between I and C1 (P<0.01). O.2) O.3) O.4) O.5) No significant differences between I and C1 or C2.

Abbreviations: US = United States, N = number of participants.

**Table 3:** Overview of the mental health studies.

Study	Setting	Participants	Intervention (I)	Control (C)	Outcomes (O)	Results
Eaton1995[30]	Veteran Administration Hospital Centre HIV/AIDS program, US.	MaleHIV-positiveveterans.	*Content:* Psychosocial structured telephone calls. *Duration:* weekly for 16 consecutive weeks, beginning the fourth week following the initial baseline assessment. *Delivered by:* trained ‘caring caller’. *N:* 9.	Group onlyreceiving theoutcome assessments.*N:* 8.	O.1) Rate of change in the General Severity Index of the Brief Symptom Inventory (psychosocial symptoms). O.2) General Severity Index mean change score. *Time points:* Baseline (two baseline assessments: at weeks one and three) and once a month for four months at weeks seven, 11, 15 and 19.	O.1) No significant difference between I and C (P = 0.2). O.2) I: 5.89 (SD = 4.97), C: 0.93 (SD = 5.82). Significant difference between I and C (P<0.05).
Heckman2007[31]	27 AIDS service organisationsin 13 statesof the US.	People livingwith HIVin ruralareas.	*Content:* Two interventions delivered by teleconference calls with six to eight participants per group. I.1) Coping improvement intervention. I.2) Information support intervention. *Duration:* eight weekly group sessions, 90 minutes each. *Delivered by:* I.1 Masters or PhD-level clinicians; I.2 Nurse practitioners or social workers. *N.I.1:* 108, *N.I.2:* 84.	Usual carewith access toall services.*N:* 107.	O.1) BDI. O.2) Symptom Checklist-90 revised. O.3) Life stressor burden Scale. O.4) PSRS, support from friends. O.5) PSRS, support from family. O.6) Barriers to Care Scale. O.7) Social well-being. O.8) Emotional well-being. O.9) Coping Self-Efficacy Scale (all O are scores). *Time points:* baseline, post-intervention, four months and eight months follow-up.	O.4) Significant difference between I.2 and C at four months (P<0.04) and eight months (P<0.05). O.6) Significant difference between I.2 and C at four months (P<0.05). O.1) O.2) O.3) O.5) O.7) O.8) O.9) No significant differences between I1, I2 and C.
Stein2007[32]	Two AIDS programclinics, Rhode IslandHospital, US.	Depressedpeople livingwith HIV.	*Content:* Family Intervention Telephone Tracking (with informal caregivers) by telephone calls. *Duration:* Weeks one to six, eight, ten, 12, 14, 18 and 22 following the initial phone call (maximum of 12 calls), before six-months interview. *Delivered by:* Eight therapists. *N*: 89.	Group onlyreceiving theoutcome assessments.*N:* 88.	O.1) BDI mean change score. O.2) Relative BDI reduction ≥50%. O.3) Categorical BDI improvement. O.4) BDI <10. O.5) Use of psychiatric medications. O.6) Mental and physical function subscales of the Short Form-36 mean change score. *Time points:* Baseline and six months after enrolment (no follow-up).	O.1) - O.6) no significant differences between I and C.
Ransom2008[33]	AIDS serviceorganisationsin ten statesof the US.	Depressedpeople livingwith HIVin ruralareas.	*Content:* Brief interpersonal therapy delivered by telephone calls. *Duration:* six 50-minutes sessions. *Delivered by:* 11 master’s-level clinical psychology trainees and one doctoral-level clinical psychologist. *N:* 41.	Usual carewith access toall services.*N:* 38.	O.1) BDI mean change score. O.2) Outcomes Questionnaire (psychiatric distress). O.3) PSRS O.4) Loneliness Scale (all O are mean change scores). *Time points:* pre- and post-intervention.	O.1) I: 5.2 (pre: 28.7, SD = 11.2 and post: 23.5, SD = 12.5), C: 0.5 (pre: 26.1, SD = 10.8 and post 25.6: SD = 13.5). Significant difference between I and C (P<0.05). O.2) I: 8.5 (pre: 87.0, SD = 24.0 and post: 78.5, SD = 31.3), C: 1.5 (pre: 78.2, SD = 22.0 and post: 76.7, SD = 26.6). Significant difference between I and C (P<0.05). O.3) O.4) No significant differences between I and C.

Abbreviations: US = United States, N = number of participants, SD  =  Standard deviation, BDI  =  Beck Depression Inventory, PSRS  =  Provision of Social Relations Scale.

**Table 4:** Overview of the medication adherence and smoking cessation studies.

Study	Setting	Participants	Intervention (I)	Control (C)	Outcomes (O)	Results
*Medication adherence*						
Collier 2005 [34]	Substudy of AIDS Clinical Trials Group study in the US. Puerto Rico and Italy.	Antiretroviral treatmentinitiating peopleliving withHIV.	*Content:* Serial supportive telephone calls focusedon medication-taking behaviour and usual adherence support. *Duration:* Initial call, then at weeks one, two, three, six, 12, and every eight weeks thereafter, as long as the participant continued to receive the study regimen with a maximum of 16 calls over 96 weeks. *Delivered by:* nurses. *N:* 142.	Usual adherence support. *N:* 140.	O.1) Virologic failure. Time points: weeks 0, eight, 16,24, 32, 40, 48, 56, 64, 72, 80, 88, 96, 104, 112, 120, 128. O.2)≥95% medication adherence. Time points: weeks 0, eight, 16, 24, 48, 72, and 96 and if participants had confirmed virologic failure.	O.1) I: 32% C: 37%. No significant difference between I and C (P = 0.32). O.2) I: 53%, C: 48% at 96 weeks. No significant difference between I and C (P = 0.46, odds ratio = 0.86, 95% confidence interval [0.57–1.29]).
Reynolds 2008 [35]	Substudy of AIDS Clinical Trials Group study in five states of the US.	Antiretroviral treatmentinitiating peopleliving withHIV.	*Content:* Serial supportive telephone calls guided by the self-regulation theory and ‘tailored to the individual and structured to address common barriers to adherence and promote effective self-care strategies’, 24-hour helpline and standard care patient education. *Duration:* 14 telephone calls from weeks one to 12, then in weeks 14, and 16. *Delivered:* registered nurse specialists. *N:* 54.	Usual care patient education. *N:* 55.	O.1) Mean adherence rate. Time-points: across weeks 4 to 64. O.2) Time to virologic failure. Time-points: weeks four, eight, 12, 16, 20, and24; and every eight weeks thereafter.	O.1) I: 97.5%; SD = 4.8, C: 97.0% SD = 5.0. Significant difference between I and C (P = 0.023). O.2) No significant difference between I and C (P = 0.21).
*Smoking cessation*						
Vidrine 2006 [36]	Primary-care clinic located within a large, inner-city HIV/AIDS care centre, Houston, Texas, US.	Smoking people living with HIV, multi-ethnic and disadvantaged.	*Content:* Proactive counselling cell phone calls focusing on increasing social support and teaching coping- and problem-solving skills, 24-hour helpline and recommended standard care. *Duration:* eight sessions in two months. *Delivered by:* trained research assistant. *N:* 48.	Recommendedusual care.*N:* 47.	O.1) Point prevalence abstinence. O.2) Sustained abstinence. O.3) Number of quit attempts. O.4) Longest period of abstinence. *Time points:* three months follow-up.	O.1) I: 29.2%, C: 8.5% (P = 0.040, odds ratio = 3.8, 95% confidence interval [1.1–13.4]). O.4) I: 24.4 days, C: 10.2 days (P = 0.023, odds ratio = 12.3, 95% confidence interval [1.7–23.0]). O.2) O.3) No significant difference between I and C.

Abbreviations: US = United States, N = number of participants, SD = standard deviation.

### Included Study Characteristics

We divided the nine identified RCTs into five intervention categories: providing HIV test results via the telephone (Table 2) [28], reducing sexual transmission risk (Table 2) [29], improving mental health (Table 3) [30], [31], [32], [33], improving medication adherence (Table 4) [34], [35] and smoking cessation (Table 4) [36]. The duration of the intervention varied from a single call to retrieve HIV test results and post-test counselling [28] to 18 weekly sessions of in-person therapy [29]. Seven studies mentioned using a theory [29], [30], [31], [32], [33], [35], [36] to develop their intervention. Two studies gave participants the option of calling a 24-hour helpline [35], [36]. Studies mainly used landline-to-landline telephone communication, except for one study which provided participants with mobile phones to receive the telephone calls [36].

All studies were published in English, and most studies were conducted in the United States only. One study was undertaken in the United States, Puerto Rico, and Italy [34]. Sample sizes varied from 17 [30] to 351 [28]. One study included only men [30], all other studies included both sexes. Two of the trials were sub-studies of larger ART adherence trials, which included ART-initiating participants [34], [35]. Other studies targeted HIV-positive smokers [36], substance using HIV-positive youth [29], homeless youth and youth at high risk of acquiring HIV [28], PLHIV in rural areas [31], [33] or depressed PLHIV [32], [33]. Participants took part individually in most studies, only one study investigated group interventions [31]. Outcomes were focussed on patients only; none of the studies reported provider-related outcomes. One study compared an intervention group with two control groups: one control receiving the intervention in person and one receiving only the assessments (the latter received the in-person intervention at the end of the intervention period) [29]. Other studies had one intervention group and one control group receiving usual care [28], [31], [33], [34], [35], [36] or only the outcome assessments [30], [32].

### Risk of Bias in Included Studies

**Figure 2:**
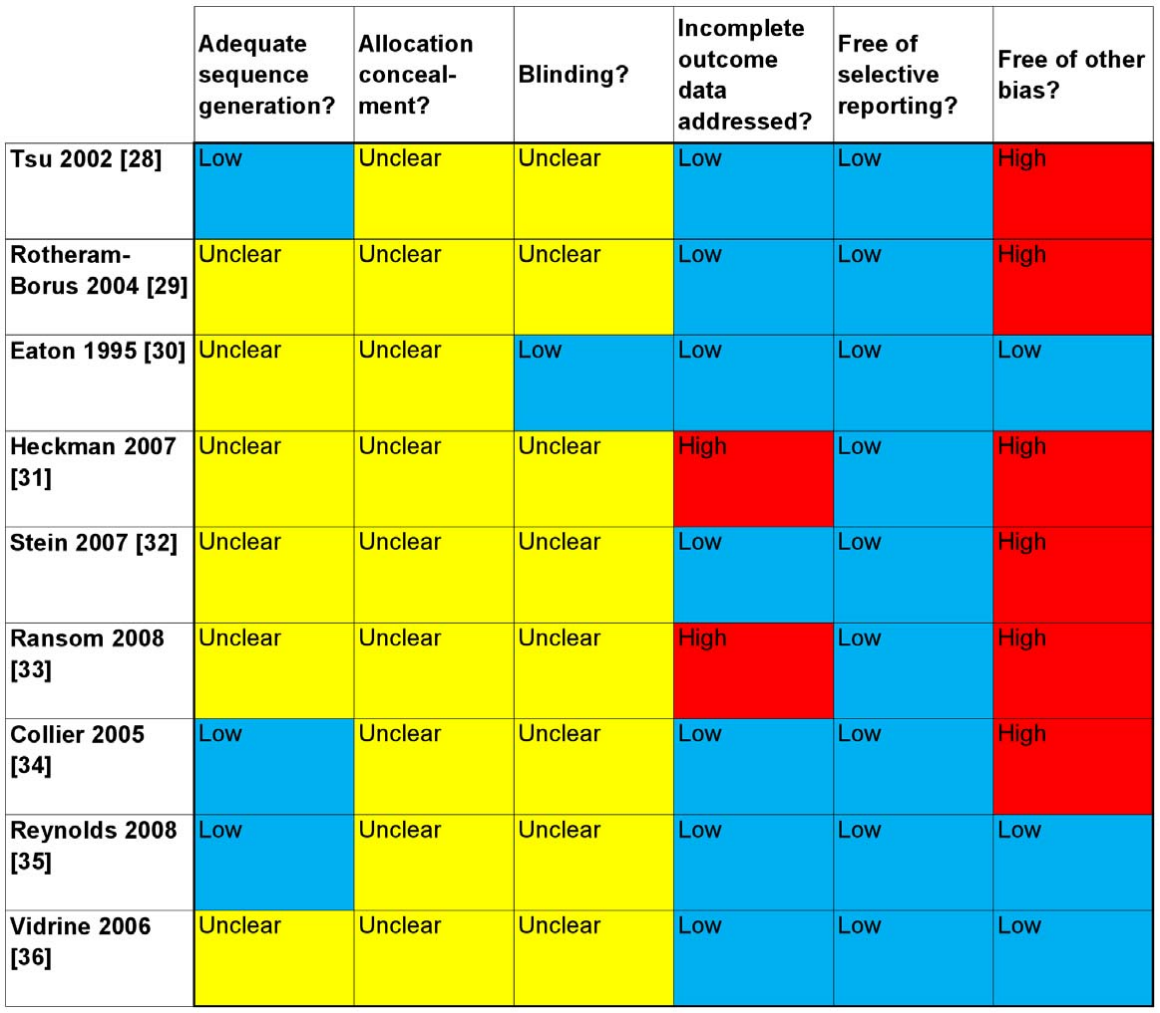
Risk of bias summary. Blue = low risk of bias; Red = high risk of bias; Yellow = unclear risk of bias. Methodological quality summary of the risk of bias; review authors’ judgements about each methodological quality item for each included study based on published articles.

Figure 2 presents a summary of the risk of bias, based on the information we found in published articles. All studies reported that participants were randomized, though only the test results study described the exact procedures used [28]. For the two medication adherence sub-studies [34], [35] we found the randomization information in their original study reports [66], [67]. None of the studies reported adequate allocation concealment. One study reported blinding the outcome assessors [30]. One study stated that all participants completed the study [30]. Seven studies gave attrition rates or numbers [29], [31], [32], [33], [34], [35], [36]. The percentages of participants who completed the last assessment were more than 80% in four studies [29], [32], [34], [36]. Five studies gave reasons for attrition [31], [33], [34], [35], [36]. Six studies reported using the intention to treat principle for analysis [29], [31], [32], [34], [35], [36]. Two studies reported using the last observation carried forward approach for missing outcomes [31], [33]. This approach can lead to serious bias, especially when the time between observations is long [26].

Studies reported all expected outcomes based on the Methods sections of papers, as we were unable to retrieve protocols from all studies for investigating this domain. Five studies reported baseline imbalance [28], [29], [32], [33], [50]. In one study, a crossover effect of the intervention may have occurred, as the usual adherence support procedures were not specified per protocol [34].

### Effects of Interventions

#### Primary outcomes

Clinical outcomes were reported in eight studies [29], [30], [31], [32], [33], [34], [35], [36], behavioural outcomes in four studies [29], [34], 35,36 and healthcare delivery-related outcomes in three studies [28], [29], [36].

Two studies reporting virologic failure did not show significant improvements [34], [35]. Five studies reported on depression and psychological distress [29], [30], [31], [32], [33]; the results were not consistent. Two articles [30], [37] related to one study [26] reported different outcomes for psychological distress based on different analyses. The first report [30] reported no significant difference in rate of change for psychosocial distress. The second report [37] showed that the intervention significantly decreased the levels of psychological distress. One study showed significantly larger reductions in depression and psychological scores in the intervention group when compared to the control group [33]. Another study found significant decreases in depression, but these did not significantly differ between the groups [32]. One study did not find a significant effect for emotional distress [29].

A study targeting homeless substance-using youth found no evidence for effectiveness of the intervention in reducing risky behaviour (sexual risk behaviour and substance use). The percentage of protected acts with HIV-negative partners significantly decreased in the telephone group compared to participants who received the intervention in-person. Other outcomes, such as medication adherence, were not significantly improved in the telephone intervention group [29]. One medication adherence study found significant improvement in adherence [35], while the other trials did not find increased adherence [29], [34]. The smoking cessation trial showed that the point prevalence abstinence and the longest period of abstinence were significantly higher in the telephone group than in the control group. There was no difference in sustained abstinence or number of quit attempts [36].

One study found that giving young people the option of receiving HIV test results by telephone significantly increased the proportion of young people receiving results. In addition, most of the telephone option group participants chose to receive their results by telephone and they received their results significantly quicker than the control group [28]. Information support intervention participants reported fewer barriers to care than usual-care participants in a study aiming to improve disease coping [31]. One study did not find evidence of effectiveness for the telephone-delivered intervention in reducing the number of missed appointments [29].

### Secondary Outcomes

Two studies reported social outcomes; information support participants received more support from friends than usual care participants in one study [31]. Another study did not report significant differences for loneliness and social support from family and friends outcomes [33]. Only one study reported the costs of the intervention; the total cost of the telephone intervention was $2692 (approximately $897 per module) per participant compared to $3500 for the in-person intervention [29]. None of the studies provided data on mortality, adverse outcomes or quality of life.

## Discussion

### Key Findings

This systematic review identified nine RCTs investigating a range of interventions in different types of participants. This heterogeneity prevented statistical pooling of the data. We found some evidence to support telephone consultation interventions for HIV/AIDS care. Giving HIV test results by telephone can increase the number of persons receiving their results and telephone support can help HIV-positive smokers quit smoking for a short period of time. The evidence for improving ART adherence and for improving mental health outcomes in participants with AIDS was not consistent. Virologic outcomes did not improve in any of the studies identified. We found no evidence of telephone calls reducing risky behaviour in youth living with HIV or those at high risk of transmission of HIV.

### Strengths and Limitations of Included Studies

Generalizability of the included studies was low as most studies were undertaken in the United States. Specific conditions further limited generalizability, for example, a medication trial reported that the medication regimes that the trial studied were more complex than most regimens used in daily practice [34]. Also, participants in the trials reported high adherence compared to the general AIDS clinic population [68]. This reduced the potential for interventions to improve outcomes and shows that study authors should carefully choose the target population of their intervention.

No studies were undertaken in a resource-limited setting. People living in these settings have extra barriers for attending care including limited financial means for travelling, fear of stigma and partner violence. We have limited knowledge about the use of mobile phones for consultation as only one study used mobile phones to deliver the intervention [36].

Although overall risk of bias in included studies was considerable, in particular for the sequence generation, allocation concealment and blinding domains, it remains unclear to what extent the study results were biased.

Intervention integrity was not well reported by all studies, this makes it harder to identify which aspects of the intervention are related to success or failure. The authors of one medication adherence study mentioned using a centralized approach for delivery of their intervention, thereby controlling for within-site dispersion and possibly also for provider bias [35], in contrast to the other adherence study in which nurses within a site delivered both the intervention and usual care [34]. In the latter study a cross-over effect of the intervention may have occurred as the usual adherence support procedures were not specified per protocol [34]. Also, the control group received telephone calls; 41% of the study sites reported making at least one telephone call to selected participants who were judged at high risk for low adherence.

Five studies reported low (<20%) loss-to-follow up, but some trials reported high percentages of participants failing to complete the intervention, which limits the potential effectiveness of the intervention. One study reported that 40% of the participants did not complete 50% or more of all intervention sessions [31], and another study reported that only 29% of the participants completed all 18 telephone sessions and 31% did not participate in any session at all [29]. High levels of attrition can be concerning given that participants are already willing to take part in trials. Studies reported giving incentives to participants, varying from 20$ US to 30$ US per assessment [30], [31], [33], or even also for intervention sessions [29]. This may increase adherence to the intervention, but may not be feasible in daily practice.

Many studies had a small number of participants [29], [30], [33], [35], [36]. For example, the study aiming to reduce sexual risk behaviour calculated that it needed 5.4 times more participants to show significance [29]. For adherence studies it has been suggested that for a study comparing one intervention group and one control group, at least 60 participants per group should be included to achieve a power of 80% and detect an absolute difference of 25% in the proportion of participants having acceptable adherence [69]. Studies assessing ART adherence used subjective questionnaires [29], [34], [35]. Self-reported outcomes are related to low sensitivity, resulting in PLHIV with low adherence not being identified. Objective measures, such as direct observation and pill counts, are more precise [70]. Some studies used objective measures: one study compared positive urine screening results and self-reported substance use [29]. The smoking cessation trial used expired Carbon Monoxide levels to biochemically verify smoking status [36]. While interventions aiming to improve long-term effects require a minimum follow-up of six months [71], only two studies fulfilled this criterion [31], [36].

### Strengths and Limitations of this Review

A strength of this review is its comprehensive search strategy aiming to identify all published and unpublished literature, thereby avoiding publication bias. We followed the high-standard systematic review methodology from the Cochrane Collaboration [26]. We only included RCTs, thus providing the highest level of evidence on effectiveness. However, as telephone consultation is a complex intervention, other types of studies such as before-and-after trials, quasi-RCTs, interrupted-time-series studies and descriptive studies could have provided additional evidence. A systematic review including these types of studies could help to bridge the gap between science and practice. Another limitation is that we chose to include a variety of interventions using telephone consultations which made comparison of data difficult.

### Comparison with Extant Literature

Evidence for effectiveness of giving test results by telephone was also found in a review comparing alternative HIV testing methods including rapid testing, oral fluid testing, home testing, and telephone post-test counselling with conventional testing. It showed that telephone post-test counselling was the second most effective intervention [72]. Another review found that telephone-administered psychotherapy interventions reduced depressive symptoms and were well received [14]. A Cochrane review showed that proactive telephone counselling helps smokers to quit [73]. Another Cochrane review showed that mobile phone based smoking cessation interventions have a positive effective in the short-term (the trial we included [36] was excluded in the Cochrane review because of short follow-up [71]), which is consistent with our findings. Smoking has additional health risks for PLHIV and increasing the effectiveness of cessation interventions is very important for improving the health of this group [74], [75], [76].

### Implications for Research and Practice

Overall, evidence of how telephone consultations fit into the current HIV/AIDS care needs further development, especially in developing countries. There are differing views on the appropriateness of trial research for studying complex interventions such as ones using communication technology [77], but future studies will require improved methodologies with a sufficient number of participants, longer follow-up and objective measurement. In addition to patient-related outcomes, provider outcomes, cost-effectiveness and adverse effects should be measured.

Video calling may possibly be increasingly used in the near future. While this might not be appropriate for individuals who experience stigmatisation because of their illness, this technology could provide the advantages of both face-to-face contact and telephone consultation. A pilot trial using this technology for improving ART adherence showed promising results [78].

The need for prevention and the large increasing number of PLHIV, who need ART and develop chronic illnesses, can have substantial consequences when remaining unaddressed. Neither national governments nor donor funding can meet the economic and health costs [2] and therefore telephone consultations can be a valuable tool for improving health outcomes of PLHIV.

This review showed that the telephone can be efficiently used for delivering HIV test results and that there is potential for telephone consultation to help PLHIV to take their medication and to quit smoking. However, this evidence of effectiveness is weak as we found only a limited number of studies. The most appropriate context for the use of this technology and key factors for its successful application in health interventions for AIDS remain unclear. The effectiveness of mobile phones in healthcare needs to be further evaluated given its expanding use and the potential mobile phones offer in resource-limited settings to reduce the HIV/AIDS burden.

## References

[1] UNAIDS (2011). Global HIV/AIDS Response: epidemic update and health sector progress towards Universal Access..

[2] AIDS2031 (2011). AIDS: Taking a long-term view: FT Press.

[3] WHO (2009). Towards universal access: scaling up priority HIV/AIDS interventions in the health sector..

[4] UNAIDS (2011). UNAIDS DATA TABLES | 2011.

[5] Ullrich A, Ott JJ, Vitoria M, Martin-Moreno JM, Atun R (2011). Long-term care of AIDS and non-communicable diseases.. Lancet.

[6] Atun R, Bataringaya J (2011). Building a durable response to HIV/AIDS: implications for health systems.. J Acquir Immune Defic Syndr.

[7] ITU-D (2011). The World in 2011 ICT Facts and Figures..

[8] Waegemann CP (2010). mHealth: the next generation of telemedicine?. Telemed J E Health.

[9] WHA (2005). WHA58.28 - eHealth Ninth plenary meeting, 25 May 2005: WHO Committee A.

[10] Car J, Sheikh A (2003). Telephone consultations.. British Medical Journal.

[11] Bunn F, Byrne G, Kendall S (2004). Telephone consultation and triage: effects on health care use and patient satisfaction..

[12] Dale J, Caramlau IO, Lindenmeyer A, Williams SM (2008). Peer support telephone calls for improving health..

[13] Roffman R (2007). Telephone-delivered interventions for people living with HIV/AIDS: Guest editorial.. Aids and Behavior.

[14] Mohr DC, Vella L, Hart S, Heckman T, Simon G (2008). The effect of telephone-administered psychotherapy on symptoms of depression and attrition: A meta-analysis.. Clinical Psychology-Science and Practice.

[15] Bunn F, Byrne G, Kendall S (2005). The effects of telephone consultation and triage on healthcare use and patient satisfaction: a systematic review.. British Journal of General Practice.

[16] Inglis SC, Clark RA, McAlister FA, Ball J, Lewinter C (2010). Structured telephone support or telemonitoring programmes for patients with chronic heart failure..

[17] Car J, Freeman GK, Partridge MR, Sheikh A (2004). Improving quality and safety of telephone based delivery of care: teaching telephone consultation skills.. Quality & Safety in Health Care.

[18] Stover J, Korenromp EL, Blakley M, Komatsu R, Viisainen K (2011). Long-Term Costs and Health Impact of Continued Global Fund Support for Antiretroviral Therapy.. PLoS One.

[19] Bos AER, Visser GC, Tempert BF, Schaalma HP (2004). Evaluation of the Dutch AIDS information helpline: an investigation of information needs and satisfaction of callers.. Patient Education and Counseling.

[20] UNAIDS (2002). HIV/AIDS Counselling, Just a Phone Call Away..

[21] McKinstry LA, Goldbaum GM, Meischke HW (2007). Telephone notification of HIV test results: Impact in king County, Washington.. Sexually Transmitted Diseases.

[22] Morrison RE, Black D (1998). Telephone medical care of patients with HIV/AIDS.. AIDS Patient Care and STDs.

[23] Albarracin D, Gillette JC, Earl AN, Glasman LR, Durantini MR (2005). A test of major assumptions about behavior change: A comprehensive look at the effects of passive and active HIV-prevention interventions since the beginning of the epidemic.. Psychological Bulletin.

[24] Fishbein M (2000). The role of theory in HIV prevention.. Aids Care-Psychological and Socio-Medical Aspects of Aids/Hiv.

[25] Reynolds NR (2003). The problem of antiretroviral adherence: a self-regulatory model for intervention.. Aids Care-Psychological and Socio-Medical Aspects of Aids/Hiv.

[26] Higgins J, Green S (2011). Cochrane Handbook for Systematic Reviews of Interventions Version 5.1.0 [updated March 2011].

[27] EndNoteX4 (2009). : Thomson Reuters.

[28] Tsu RC, Burm ML, Gilhooly JA, Sells CW (2002). Telephone vs. face-to-face notification of HIV results in high-risk youth.. J Adolesc Health.

[29] Rotheram-Borus MJ, Swendeman D, Comulada WS, Weiss RE, Lee M (2004). Prevention for substance-using HIV-positive young people: telephone and in-person delivery.. J Acquir Immune Defic Syndr.

[30] Eaton AH (1995). Immunological and psychological effects of “TELECARE” in HIV seropositive males: Lymphocyte subpopulations, cortisol, neopterin, and psychosocial factors: Fuller Theological Seminary, School of Psychology, US.

[31] Heckman TG, Carlson B (2007). A randomized clinical trial of two telephone-delivered, mental health interventions for HIV-infected persons in rural areas of the United States.. AIDS Behav.

[32] Stein MD, Herman DS, Bishop D, Anderson BJ, Trisvan E (2007). A telephone-based intervention for depression in HIV patients: negative results from a randomized clinical trial.. AIDS Behav.

[33] Ransom D, Heckman TG, Anderson T, Garske J, Holroyd K (2008). Telephone-delivered, interpersonal psychotherapy for HIV-infected rural persons with depression: a pilot trial.. Psychiatr Serv.

[34] Collier AC, Ribaudo H, Mukherjee AL, Feinberg J, Fischl MA (2005). A randomized study of serial telephone call support to increase adherence and thereby improve virologic outcome in persons initiating antiretroviral therapy.. J Infect Dis.

[35] Reynolds NR, Testa MA, Su M, Chesney MA, Neidig JL (2008). Telephone support to improve antiretroviral medication adherence: a multisite, randomized controlled trial.. J Acquir Immune Defic Syndr.

[36] Vidrine DJ, Arduino RC, Lazev AB, Gritz ER (2006). A randomized trial of a proactive cellular telephone intervention for smokers living with HIV/AIDS.. Aids.

[37] Lucy JR (1995). The effects of telecare on psychosocial symptoms in hiv-seropositive individuals: Fuller Theological Seminary, School of Psychology, US.

[38] Ransom D (2007). Telephone-delivered, interpersonal therapy for HIV-infected rural persons with depression: A pilot randomized clinical trial [3280036]..

[39] Herman DS, Bishop D, Anthony JL, Chase W, Trisvan E (2006). Feasibility of a telephone intervention for HIV patients and their informal caregivers.. Journal of Clinical Psychology in Medical Settings.

[40] Vidrine DJ, Arduino RC, Gritz ER (2006). Impact of a cell phone intervention on mediating mechanisms of smoking cessation in individuals living with HIV/AIDS.. Nicotine Tob Res.

[41] Cosio D, Heckman TG, Anderson T, Heckman BD, Garske J (2010). Telephone-administered motivational interviewing to reduce risky sexual behavior in HIV-infected rural persons: a pilot randomized clinical trial.. Sex Transm Dis.

[42] Bartholow BN (2006). A comparison of consumer-controlled and traditional HIV counseling and testing: Implications for screening and outreach among injection drug users: Georgia State University, US.

[43] Chiou P, Kuo BI, Lee M, Chen Y, Wu S (2004). A program of symptom management for improving self-care for patients with HIV-AIDS.. AIDS Patient Care & STDs.

[44] Cook PF, McCabe MM, Emiliozzi S, Pointer L (2009). Telephone nurse counseling improves HIV medication adherence: an effectiveness study.. J Assoc Nurses AIDS Care.

[45] Cosio D (2008). A telephone-delivered, motivational interviewing intervention to reduce risky sexual behavior in HIV-infected rural persons: A pilot randomized clinical trial [3327154]..

[46] Ellen JM, Gurvey JE, Pasch L, Tschann J, Nanda JP (2002). A randomized comparison of A-CASI and phone interviews to assess STD/HIV-related risk behaviors in teens.. J Adolesc Health.

[47] Fife BL, Scott LL, Fineberg NS, Zwickl BE (2008). Promoting adaptive coping by persons with HIV disease: evaluation of a patient/partner intervention model.. JANAC: Journal of the Association of Nurses in AIDS Care.

[48] Frank AP, Wandell MG, Headings MD, Conant MA, Woody GE (1997). Anonymous HIV testing using home collection and telemedicine counseling. A multicenter evaluation.. Arch Intern Med.

[49] Heckman TG, Anderson ES, Sikkema KJ, Kochman A, Kalichman SC (2004). Emotional distress in nonmetropolitan persons living with HIV disease enrolled in a telephone-delivered, coping improvement group intervention.. Health Psychology.

[50] Heckman TG, Barcikowski R, Ogles B, Suhr J, Carlson B (2006). A telephone-delivered coping improvement group intervention for middle-aged and older adults living with HIV/AIDS.. Ann Behav Med.

[51] McKinstry LA, Goldbaum GM, Meischke HW (2007). Telephone notification of HIV test results: Impact in king County, Washington.. Sexually Transmitted Diseases.

[52] Picciano JF, Roffman RA, Kalichman SC, Rutledge SE, Berghuis JP (2001). A telephone based brief intervention using motivational enhancement to facilitate HIV risk reduction among MSM: a pilot study.. AIDS & Behavior.

[53] Puccio JA, Belzer M, Olson J, Martinez M, Salata C (2006). The use of cell phone reminder calls for assisting HIV-infected adolescents and young adults to adhere to highly active antiretroviral therapy: a pilot study.. AIDS Patient Care STDS.

[54] Roffman RA, Picciano JE, Ryan R, Beadnell B, Fisher D (1997). HIV-prevention group counseling delivered by telephone: an efficacy trial with gay and bisexual men..

[55] Spielberg F, Critchlow C, Vittinghoff E, Coletti AS, Sheppard H (2000). Home collection for frequent HIV testing: acceptability of oral fluids, dried blood spots and telephone results.. Aids.

[56] Wang H, Zhou J, Huang L, Li X, Fennie KP (2010). Effects of nurse-delivered home visits combined with telephone calls on medication adherence and quality of life in HIV-infected heroin users in Hunan of China.. J Clin Nurs.

[57] Wewers ME, Neidig JL, Kihm KE (2000). The feasibility of a nurse-managed, peer-led tobacco cessation intervention among HIV-positive smokers.. J Assoc Nurses AIDS Care.

[58] Wu AW, Jacobson DL, Berzon RA, Revicki DA, van der Horst C (1997). The effect of mode of administration on medical outcomes study health ratings and EuroQol scores in AIDS.. Qual Life Res.

[59] Konkle-Parker DJ, Erlen JA, Dubbert PM (2010). Lessons learned from an HIV adherence pilot study in the Deep South.. Patient Education and Counseling.

[60] Quinones C, Boyle BA (2004). A simple method to improve adherence to office visits: appointment reminder calls..

[61] Konkle-Parker DJ (2002). Tailored adherence interventions for HIV-infected adults based on the transtheoretical model of change..

[62] Muko K, Chingang L, Yenwong M (2009). Use of mobile phones to improve on adherence and clinical outcome of patients on ART..

[63] Cox HL, Johnson JW, Williamson JC, Russell GB, Wilkin AM (2006). Clinician-initiated telephone follow-up improves virologic outcomes in an HIV outpatient clinic..

[64] Reynolds NR, Alonzo AA, Nagaraja HN (2008). A telephone-delivered adherence intervention improves clinical outcomes..

[65] Moher D, Liberati A, Tetzlaff J, Altman DG, Grp P (2009). Preferred reporting items for systematic reviews and meta-analyses: the PRISMA statement.. British Medical Journal.

[66] Smeaton LM, DeGruttola V, Robbins GK, Shafer RW (2001). ACTG (AIDS Clinical Trials Group) 384: a strategy trial comparing consecutive treatments for HIV-1.. Control Clin Trials.

[67] Fischl MA, Ribaudo HJ, Collier AC, Erice A, Giuliano M (2003). A randomized trial of 2 different 4-drug antiretroviral regimens versus a 3-drug regimen, in advanced human immunodeficiency virus disease.. J Infect Dis.

[68] Reynolds NR (2004). Adherence to antiretroviral therapies: State of the science.. Current Hiv Research.

[69] McDonald HP, Garg AX, Haynes RB (2002). Interventions to enhance patient adherence to medication prescriptions - Scientific review.. Jama-Journal of the American Medical Association.

[70] Rueda S, Park-Wyllie LY, Bayoumi AM, Tynan AM, Antoniou TA (2006). Patient support and education for promoting adherence to highly active antiretroviral therapy for HIV/AIDS..

[71] Whittaker R, Borland R, Bullen C, Lin RB, McRobbie H (2009). Mobile phone-based interventions for smoking cessation..

[72] Hutchinson AB, Branson BM, Kim A, Farnham PG (2006). A meta-analysis of the effectiveness of alternative HIV counseling and testing methods to increase knowledge of HIV status.. Aids.

[73] Stead LF, Perera R, Lancaster T (2006). Telephone counselling for smoking cessation..

[74] Kwong J, Bouchard-Miller K (2010). Smoking Cessation for Persons Living With HIV: A Review of Currently Available Interventions.. Janac-Journal of the Association of Nurses in Aids Care.

[75] Vidrine DJ (2009). Cigarette smoking and HIV/AIDS: health implications, smoker characteristics and cessation strategies.. AIDS Educ Prev.

[76] Nahvi S, Cooperman NA (2009). Review: the need for smoking cessation among HIV-positive smokers.. AIDS Educ Prev.

[77] Puskin DS, Cohen Z, Ferguson AS, Krupinski E, Spaulding R (2010). Implementation and evaluation of telehealth tools and technologies.. Telemed J E Health.

[78] Skrajner M, Heckman T, Camp (2009). Use of videophone technology to address medication adherence issues in persons with HIV..

